# A large measles outbreak in Zimbabwe related to vaccine hesitancy in 2022

**DOI:** 10.11604/pamj.2026.53.59.48564

**Published:** 2026-02-04

**Authors:** Nyaradzai Chiwara, Emma Tshuma, Clara Mashiringo, Raiva Simbi, Arnold Mukaratirwa, Annie Shonhai, Regis Katsande, Maxwell Rupfutse, Charles Byabamazima, Desta Tiruneh, Balcha Masresha

**Affiliations:** 1 Ministry of Health and Child Care, Harare, Zimbabwe,; 2 World Health Organization, Regional office for Africa, Brazzaville, Congo,; 3 World Health Organization, Country office for Zimbabwe, Harare, Zimbabwe,; 4 World Health Organization, Intercountry support team for Eastern and Southern Africa, Harare, Zimbabwe

**Keywords:** Zimbabwe, measles, outbreak, vaccine hesitancy, Apostolic sect

## Abstract

**Introduction:**

Zimbabwe has been implementing measles control strategies since 1998 and has adopted the regional goal for the elimination of measles and rubella by 2030. The country was one of the countries affected by a large resurgence of measles in 2010.

**Methods:**

we reviewed immunization coverage and disease surveillance data for 2014-2023 and conducted detailed analysis of the epidemiological characteristics of the measles outbreak in 2022.

**Results:**

Zimbabwe had MCV1 and MCV2 national-level coverage of 90% and 77%, respectively in 2023. A total of 4891 confirmed measles cases and 411 deaths were reported in 2022, with a case fatality ratio of 18% among children under 5 years of age. Manicaland province had the highest incidence. Age-specific incidence rates of 678 and 649 per million were documented among children aged 1-4 years and 5-9 years, respectively. The outbreak, which was linked to a national gathering of vaccine-hesitant religious groups, declined sharply in September 2022 following a nationwide vaccination campaign. Zimbabwe has maintained relatively high vaccination coverage in the years since 2010 and succeeded in reducing annual measles incidence levels to less than one case per million population. The large outbreak of 2022 is evidence of underlying risks, especially among vaccine-hesitant groups.

**Conclusion:**

the recurrence of measles outbreaks among the same religious community and the high case fatality rate deserve programmatic focus to prevent future outbreaks. A robust advocacy and communication effort should be implemented targeting Apostolic community leaders and members. In addition, it is important to tailor the provision of routine immunisation services to members of this hesitant group.

## Introduction

Zimbabwe is administratively divided into 10 provinces (8 rural and 2 metropolitan areas) and 63 districts. The total population according to the census done in 2022 was 15,178,957 [1]. Health services are delivered in 1850 health facilities spread across the country [2]. As of 2022, the infant mortality rate was 34.6 per 1000 live births, while the under-5 child mortality was 47.7 per 1000 live births [3]. The Zimbabwe Expanded Program on Immunization aims at reducing vaccine-preventable diseases such as pneumonia, diarrhea and measles, which are among the leading causes of mortality in infants and children less than 5 years of age. Vaccination services are offered daily in all health facilities, integrated with other interventions such as vitamin A supplementation and growth monitoring.

The first dose of measles vaccine (MCV1) has been provided at 9 months of age since 1981. In 2015, the second dose of measles vaccine (MCV2) was introduced as a combined Measles-Rubella (MR) vaccine, and is administered at 18 months of age. Since the first quarter of 2024, Zimbabwe has stopped using 10-dose vials and adopted the 5-dose presentation for the MR vaccine for routine immunisation services, in order to minimize wastage and reach more children [2]. The country has a vaccination catch-up policy for children under 5 years of age through the routine immunisation program [4]. The high attrition rate of health professionals is one of the major challenges for the national vaccination program [5]. Based upon religious grounds, some vaccine-hesitant groups also continue to pose challenges [6].

Zimbabwe has periodically implemented measles supplemental immunization activities (also referred to as preventive mass vaccination campaigns) starting in 1998 [7]. The introduction of the rubella vaccine was implemented through a wide age range catch-up SIA in 2015 and attained a coverage of 96% by survey [8]. The follow-up supplemental immunization activities (SIAs) in 2019 attained administrative coverage of 94% while the post-campaign survey had a coverage of 79% [8,9]. As part of the efforts towards polio eradication, Zimbabwe has been implementing surveillance for Acute Flaccid Paralysis (AFP) since the early 1990s. The national measles-rubella surveillance system in the country has been integrated with AFP surveillance implementation processes and infrastructure since 1998.

In 2010, a large outbreak of measles occurred in Zimbabwe, and was part of a wider resurgence of measles that affected 7 countries in Southern Africa, starting in 2009 in South Africa before spreading to neighboring countries. In Zimbabwe, a total of 9762 suspected cases and 8924 confirmed cases were reported at the time. Surveillance data showed that 44% of the cases were less than 5 years of age, and 23% were aged 5-9 years [10,11]. In 2022, Zimbabwe experienced another large outbreak. This manuscript reviews the measles programmatic data and the epidemiological information related to the 2022 outbreak.

## Methods

We reviewed the WHO/UNICEF estimates of immunization coverage for the first and second doses of measles vaccine in Zimbabwe for the 10 years from 2014 to 2023, and the latest Demographic Health Survey (DHS) data from 2023/2024 [12,13]. We analyzed the measles case-based surveillance and laboratory databases shared by the country with WHO [10]. For this study, we also compared the case-based surveillance information with aggregate data captured through the district health information system (DHIS-2).

The country implements a case-based surveillance system for measles as per the standard WHO African Regional measles surveillance processes, case definitions and performance indicators [14]. The surveillance system is supported by laboratory confirmation whereby suspected measles cases are investigated, serum specimens are collected and sent to the accredited national measles-rubella serological laboratory hosted in the National Virology Research Laboratory (NVRL) for IgM testing. Nasopharyngeal swabs were collected and sent to NVRL under reverse cold chain for subsequent transportation to the Regional Reference Laboratory (in the South African National Institute for Communicable Diseases) for genotyping. The measles genotyping was done using the polymerase chain reaction (PCR) method according to the WHO-recommended protocol [14].

Disease surveillance performance is monitored at the national level using standard performance indicators, including the non-measles febrile rash illness rate, and the proportion of districts that investigate at least one suspected measles case with a blood specimen per year.

## Results

**Figure 1:**
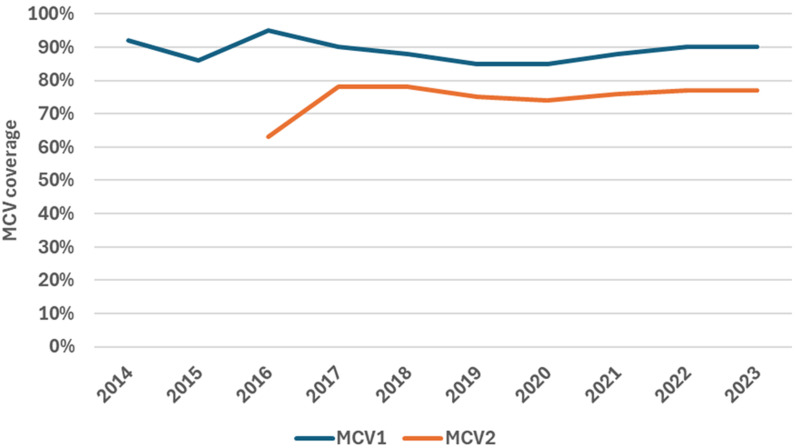
MCV1 and MCV2 coverage in Zimbabwe, WHO-UNICEF coverage estimates, 2014 - 2023

According to the WHO UNICEF estimates of vaccination coverage, Zimbabwe had MCV1 coverage of 90% and MCV2 coverage of 77% at the national level in 2023. The country experienced a 3% decline of MCV coverage in 2019 and 2020 as compared to the preceding two years but has managed to recover the coverage levels by 2022 (Figure 1). The DHS survey done in 2023 showed MR1 coverage of 84.5% at the national level, with provincial coverage ranging from 77.7% in Manicaland to 96.4% in Matabeleland North. The MR2 coverage, according to the DHS, was 70.5% at the national level.

According to the case-based measles surveillance data, Zimbabwe reported very few confirmed cases of measles in the years 2015-2021. During this time period, the national measles-rubella surveillance performance met the targets for the two principal performance monitoring indicators every year except in 2020, when the district reporting was short of the 80% target, and in 2021, when both targets were missed (Table 1).

**Table 1:** confirmed measles cases and measles surveillance performance, Zimbabwe, 2014-2023

Years	Number of confirmed measles cases	Non-measles febrile rash illness rate (target: >2.0/100,000 population)	% districts reporting at least 1 suspected measles case/ year (Target > 80%)
2014	66	15.5	100%
2015	1	2.5	86%
2016	2	3.1	94%
2017	4	4.9	92%
2018	1	3.9	91%
2019	3	2.8	92%
2020	4	1.9	92%
2021	0	1.5	71%
2022	4891	8.5	100%
2023	18	5.6	91%

In 2022, Zimbabwe reported a total of 6457 suspected measles cases, out of which 4891 cases were confirmed. A total of 4596 serum samples were received at NVRL during the outbreak, of which 2633 were tested serologically. Of these, 541 cases were confirmed by laboratory (positive for measles IgM) while 4350 cases were confirmed by epidemiological linkage. The measles outbreak started amongst members of the Johanne Marange Apostolic sect in Zindi area of Mutasa district (Manicaland Province), which was hosting a church gathering during the first week of April 2022. Specimens collected during the second week of April 2022 from 6 suspected measles cases resulted in two cases being confirmed to be measles by laboratory testing. More confirmed cases were reported in subsequent weeks. A larger gathering of the church was held from 8^th^ to 18^th^ July 2022 at Mafararikwa area in Mutare District of Manicaland Province, drawing thousands of congregants from all over the country and from neighboring countries.

In the months between May and August, more and more districts across the country were reporting confirmed measles outbreaks. Seven of the ten provinces were eventually affected, while Bulawayo, Mashonaland Central and Matabeleland South had no or very few confirmed cases. The monthly peak was reached in August 2022. Manicaland province had the highest number with a total of 2854 confirmed measles cases throughout the year (Table 2).

**Table 2:** monthly trends of confirmed measles cases by province, Zimbabwe, 2022

Province	Jan	Feb	Mar	Apr	May	Jun	Jul	Aug	Sep	Oct	Nov	Dec	Total
Bulawayo	0	0	0	0	0	0	0	0	0	0	0	0	**0**
Harare	1	2	3	5	5	4	37	79	36	0	0	0	**172**
Manicaland	4	0	14	184	178	159	693	1593	29	0	0	0	**2854**
Mashonaland Central	0	0	0	0	0	1	5	0	0	0	0	0	**6**
Mashonaland East	0	0	0	0	0	1	10	47	0	0	0	0	**58**
Mashonaland West	7	7	15	15	33	13	58	620	114	0	0	0	**882**
Masvingo	0	0	1	0	2	4	28	106	11	0	0	0	**152**
Matabeleland North	0	0	0	0	0	0	2	63	0	0	0	0	**65**
Matabeleland South	0	0	3	1	5	1	5	7	9	0	0	0	**31**
Midlands	0	0	0	0	0	0	0	488	183	0	0	0	**671**
**Total**	**12**	**9**	**36**	**205**	**223**	**183**	**838**	**3003**	**382**	**0**	**0**	**0**	**4891**

More than half (58%) of the total confirmed cases were from Manicaland province, with Buhera district accounting for 34% of all cases. The overall annual incidence of confirmed measles at national level was 322 cases per million population. The highest provincial incidence level was in Manicaland province with 1401 cases per million population (Table 3). The ages of 774 confirmed cases were not documented. The largest number of cases was reported in the age group less than 5 years (1656 cases making up 40%), followed by 1498 cases (36%) in the age group 5-9 years. Nearly a fifth (18%) of the cases were aged 10-14 years (Table 4).

**Table 3:** provincial incidence of confirmed measles, Zimbabwe, 2022

Province	Total population	Number of confirmed measles cases (Percentage)	Incidence of confirmed measles per million population
Bulawayo	665,952	0 (0%)	0
Harare	2,427,231	172 (3.5%)	71
Manicaland	2,037,703	2854 (58%)	1,401
Mashonaland Central	1,384,891	6 (0.1%)	4
Mashonaland East	1,731,173	58 (1.2%)	34
Mashonaland west	1,893,584	882 (18%)	466
Matabeleland North	827,645	65 (1.3%)	79
Matabeleland South	760,345	31 (0.6%)	41
Masvingo	1,638,528	152 (3.1%)	93
Midlands	1,811,905	671 (13.7%)	370
**Total**	**15,178,957**	**4891 (100%)**	**322**

**Table 4:** age specific measles incidence rate, Zimbabwe, 2022

Age group in years	Population	Confirmed measles cases	Age-specific incidence rate/million population
<1 year	539,353	267	495
1 to 4 years	2,047,845	1389	678
5 to 9 years	2,307,585	1498	649
10 to 14 years	2,043,634	757	370
15 years and above	10,042,096	206	21

The vaccination status of the cases is missing in 4065 of the 4891 confirmed cases. Among the 828 cases with recorded vaccination status, 84% were unvaccinated, while 4% have received one dose and 7% claimed to have received two or more doses. Four percent of the cases were reported as unknown vaccination status. There were 411 deaths reported in the case-based surveillance database, with 77% occurring among children aged less than 5 years. The highest age-specific case fatality ratio was recorded among children less than 5 years of age (Table 5).

**Table 5:** age specific case fatality ratios, Zimbabwe, 2022

Age group	Number of measles deaths	Number of confirmed measles cases (%)	Age specific case fatality ratio
< 1 year	48 (11.7%)	267 (5.5%)	18%
1 - 4 Years	267 (65%)	1389 (28.4%)	19%
5 - 9 years	51 (12.4%)	1498 (30.6%)	3%
10 - 14 years	23 (5.6%)	757 (15.5%)	3%
15 years plus	6 (1.5%)	206 (4.2%)	3%
unknown	16 (3.9%)	774 (15.8%)	
Total	411 (100%)	4891 (100%)	8%

In the case-based surveillance database, information on religion is not collected. However, the district health information system (DHIS-2), which includes aggregate surveillance reporting, shows that the largest number of measles cases in 2022 (3065 cases among 5173 in the database, accounting for 59%) was among the followers of the Johane Marange Apostolic sect, while there were 989 cases (19%) among other Apostolic sects. Catholics and Methodists accounted for 83 and 42 cases respectively, and other religions accounted for 132 cases. Religious affiliation was unknown in 862 (17%) cases. As part of the case-based investigation of outbreaks, throat swab specimens collected from the lab confirmed measles cases were sent to the National Institute of Communicable Diseases (NICD) in South Africa for genotyping of viral strains. Six of the 9 throat swab specimens from Mutasa district were reported to contain the D8 strain of measles virus, while 3 were negative.

Following limited-scale outbreak response vaccination activities in some districts, Zimbabwe implemented a nationwide measles supplemental immunization activity (SIA) from 27^th^ August to 1^st^ September 2022 targeting children from 6 to 59 months of age. This was a previously scheduled follow-up SIA integrated with vitamin A supplementation that the country was preparing to implement since May 2022. The SIA was limited to the under-5 target age group due to prior funding approval from the GAVI Alliance as a preventive campaign, and due to a lack of additional resources to go beyond 5 years of age within the emergency context of the response to the large outbreak. A total of 1,970,123 children were vaccinated, giving an administrative coverage of 86% and the post-campaign coverage survey showed 90% coverage at the national level. Provincial performance ranged from 79% to 94% during the coverage survey.

## Discussion

The 47 countries in the African Region of the WHO have made progress in decreasing measles disease burden in the last three decades. The latest published estimates show that, between the years 2000 and 2023, the annual estimated number of measles cases in the African Region has declined by 58%, while the estimated number of deaths has decreased by 79%, with a total of 20.9 million measles deaths having been averted during these 23 years [15]. However, countries continue to experience measles outbreaks, most of which are limited in size and duration [16]. The 2022 outbreak in Zimbabwe was large and disruptive in size. It lasted about 4 months and was contained following the implementation of a nationwide vaccination campaign. On the other hand, during the 2010 outbreak in Zimbabwe, large clusters of cases continued to occur for more than 7 months, partly because the initial reactive vaccination campaigns were of limited scale, and there was a significant proportion of persons in the school-age group and adults affected [11].

Zimbabwe has maintained relatively high MCV1 and MCV2 coverage and implemented periodic SIAs every 3-4 years. In the 7 years between 2014 and 2021, the country had annual measles incidence levels of less than one case per million population, in the presence of good-quality surveillance performance. However, the large outbreak of 2022 is evidence of underlying risks, especially among vaccine-hesitant groups. The DHS data from 2023 showed that Manicaland province had the lowest MCV1 coverage at 77.7%, which may explain the large number of measles cases reported from the province during this outbreak. The confirmed measles cases declined sharply in September 2022, immediately following the vaccination campaign at the end of August. However, the fact that there were zero confirmed cases throughout the country in the months of October-December 2022 may indicate some challenges with case investigation and/or data sharing. Zimbabwe did not document any measles outbreak throughout 2023 and 2024.

Among the neighboring countries, Botswana experienced an uptick of confirmed measles cases between January and May 2023, with a total of 63 confirmed measles cases reported throughout the year. Twenty-three of these cases were detected in the North-East district, which borders Zimbabwe, suggesting possible importation. However, genotyping was not done to confirm this linkage [10]. In 2020, Zimbabwean migrants accounted for 58% of all migrants in Botswana [17]. Studies have shown considerable variation in the levels of acceptance of immunization services among different apostolic groups [18]. The 2019 post-measles SIAs coverage survey found that among children who missed the SIAs doses, religious objection was the most frequent reason in 16% of the respondents [9]. In a study that analyzed the Zimbabwe DHS data from 2011, it was found that Apostolic children were twice as likely to have received no basic vaccines as compared to the non-Apostolic children [19].

A sociological study documented that Apostolic members strongly believe in faith healing, and some sects exercise strict moral proscriptions against modern medicines and medical services even at the brink of death, as a demonstration of faith [20]. Even though the national immunization program has been engaging with the Apostolic religious community since the outbreak in 2010, it was evident that there was a large accumulation of susceptible children since then, partly explaining the high proportion of cases among this group. In this context, it may not be easy to promote vaccination within this group using the usual social mobilization approaches and tools. In response to the 2010 outbreak, high-level political leaders held consultations with Apostolic religious leaders, and organized a National Consultative Conference on Child Health, which created an opportunity for continuous dialogue, as well as built some trust and consensus at the time, and helped Apostolic communities to get their children vaccinated [18] However, such efforts should be maintained to avoid the accumulation of unvaccinated cohorts.

The 2022 measles outbreak was characterized by a very high case fatality ratio (CFR) of 18%-19% among children younger than 5 years of age. According to the records at the time, most of the deaths occurred in community settings. Some sects of the Apostolic community are hesitant to receive curative medical care, and this may have contributed to the high case fatality. A systematic review and modelling study on measles CFRs produced modelling estimates for low- and middle-income countries of 3% measles CFR for children under 1 year, and 1.6% CFR for those aged 1-4 years of age for the year 2019. The CFR during the Zimbabwe outbreak was very high compared to these estimates [21]. Between 2011 and 2021, the Southern African subregion managed to avert large outbreaks like the one in 2010, with the exception of Namibia in 2014 and Madagascar in 2019. The large outbreak in Madagascar appeared after many years of low incidence, which mirrors the Zimbabwean situation. However, Madagascar´s MCV1 coverage was much lower than Zimbabwe´s (ranging from 55-68% throughout the 10 years before the outbreak), 22% of cases were in the age group above 15 years, and vaccine hesitancy was not a major factor [22].

The World Health Organization recommends that every outbreak investigation be accompanied by a root cause analysis in order to understand the interplay of the factors, and the root causes associated with measles outbreaks [23]. Even though non-vaccination among vaccine-hesitant groups appears to be the major factor in the Zimbabwe outbreak, every outbreak and every district has peculiar contextual factors, and so it is likely that other factors contributed to the occurrence of the outbreak. Identifying the root causes helps immunization program managers to address them with evidence-based strategies tailored to the local context. This study has some limitations. The reporting of surveillance data may not be complete, as shown by the discrepancy between the DHIS2 and the case-based surveillance data. The case-based surveillance investigation process does not continuously follow confirmed cases to determine their outcome in the weeks after the measles diagnosis, and so cannot fully capture information on measles complications and deaths. A significant proportion of cases did not have information on vaccination status.

## Conclusion

In order to avert another outbreak in the future, a robust advocacy, communication and social mobilization effort is required to promote vaccination with the Apostolic community leaders and members. In addition, the Zimbabwean national immunization program and local partners should develop creative mechanisms of tailoring the provision of routine immunisation services to address the community´s concerns. In the planning of preventive and outbreak response campaigns, we recommend tailoring the interventions to the target populations based on local epidemiological data as needed, to reduce susceptibility among age groups where significant measles circulation occurs. Moreover, preventive SIAs should be scheduled to be implemented during the months preceding the high measles transmission period and taking into consideration the timelines for planned congregation of Apostolic communities.

### What is known about this topic

- Zimbabwe experienced a large measles outbreak in 2010 fueled by vaccine hesitancy among the Apostolic religious sect members;
- Zimbabwe has maintained relatively high vaccination coverage and has provided two doses of measles vaccine since 2015.

### What this study adds

- Vaccine-hesitant groups were mostly affected in the 2022 measles outbreak in Zimbabwe;
- The age-specific incidence and case fatality rate were highest among children below 5 years of age, with the CFR being 18% in infants and 19% among 1-5 year-olds;
- It is critical for the immunisation program and public health leaders to engage regularly with the Apostolic sect leaders and members in order to ensure that continued hesitancy does not erode the success the country has attained towards measles elimination.
